# Detection of critical transition states in complex diseases based on distance correlation coefficient

**DOI:** 10.1371/journal.pone.0341473

**Published:** 2026-07-16

**Authors:** Pingjun Hou, Changchun Liu, Xinlin Zhang

**Affiliations:** School of Mathematics and Statistics, Henan University of Science and Technology, Luoyang, China; The University of Texas, MD Anderson Cancer Center, UNITED STATES OF AMERICA

## Abstract

During the development of many complex diseases, biological systems may pass through an unstable critical transition state before disease onset or further deterioration. Timely detection of this state is important for identifying early warning signals and for supporting intervention before marked disease progression. This study proposes a model-free single-sample method based on the distance correlation coefficient, namely dCor-LNWD, for assessing disease-associated perturbations of individual diseased samples. This method uses distance correlation to evaluate both linear and nonlinear associations between gene-expression levels. This study applied dCor-LNWD to four stage-stratified cancer datasets (ESCA, KIRC, KIRP, and LUAD) from the TCGA database and the GSE13268 dataset from the GEO database, and successfully identified critical transition states in five complex disease datasets, including stage-wise critical states during cancer progression.

## Introduction

**Fig 1:**
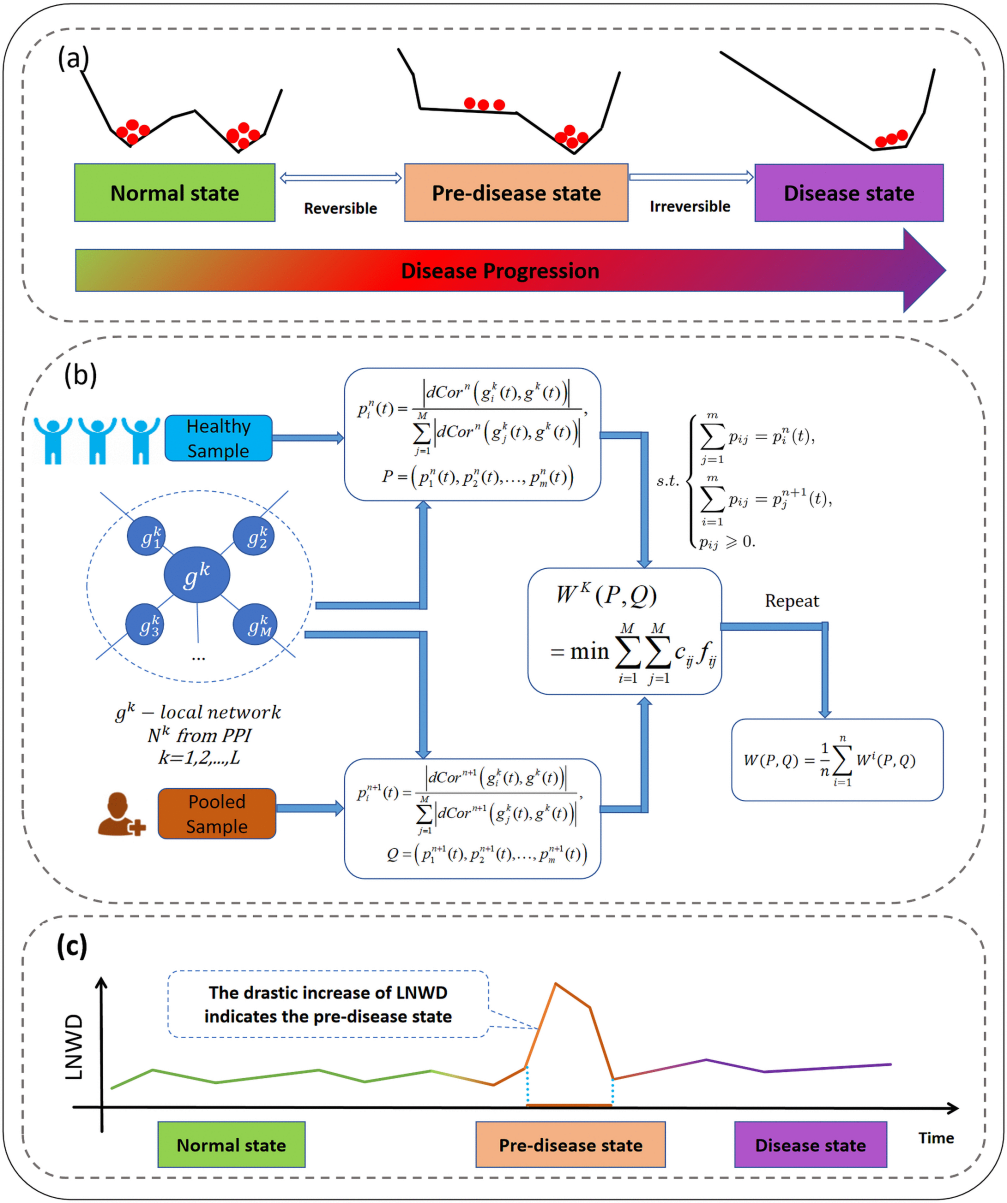
Subfigure (a) corresponds to the three stages of complex disease progression: the vertical axis represents the hypothetical potential function, a theoretical model for describing the dynamic stability of biological systems. Subfigure (b) presents the flowchart of the dCor-LNWD algorithm. Subfigure (c) identifies the critical state of the disease by means of the variation trend of dCor-LNWD scores.

The progression of many complex diseases such as cancer [1], influenza [2], and diabetes [3] is not a smooth linear process, but rather undergoes sudden and large-scale state transitions at specific stages, namely “critical transitions.” This phenomenon indicates that the evolutionary process of diseases can be generally divided into three key stages: normal state, critical state, and disease state [4–6]. The normal state is a relatively healthy and stable state; in this stage, the biological system has high robustness against external disturbances and can maintain its own stability. The critical state is the limit of the normal state, characterized by low stability and thus high disease susceptibility; most importantly, the critical state is a reversible stage—if appropriate medical intervention is applied at this moment, the system may return to the normal state. After the system crosses the critical point, it enters a new and stable state, i.e., the disease state. Similar to the normal state, it also has high stability, but this stability instead makes its progression difficult to reverse and it is usually considered almost irreversible (see Fig 1(a)). Therefore, accurately identifying this key early warning window before the catastrophic deterioration of the disease is of crucial clinical significance for seizing valuable intervention opportunities and preventing or delaying the disease progression. However, due to the inherent high nonlinearity and complexity of biological systems, effectively identifying this critical state is extremely challenging in practice.

At present, the Dynamic Network Biomarker (DNB) theoretical framework proposed by researchers provides an effective theoretical method for solving the problem of detecting the critical state of complex diseases [7]. This framework regards the development process of complex diseases as a high-dimensional nonlinear dynamic system. When the biological system approaches the pre-disease critical state, a group of dominant molecules emerges in its molecular network, and the expression levels of these molecules undergo significant differences [8]. Based on this characteristic, by identifying this group of dominant molecules to construct statistical indicators, it is possible to accurately capture the early warning signals of the arrival of the critical state of complex diseases, providing a key basis for the early intervention of diseases.

In the classical DNB framework, the pre-disease state denotes an unstable critical state before disease onset or before marked disease deterioration. In stage-stratified cancer datasets, however, the analyzed tumor samples have already been clinically diagnosed. Therefore, when applying dCor-LNWD to TCGA cancer cohorts, the identified state is interpreted as a critical state during cancer progression rather than a cancer-free pre-disease condition before tumor initiation.

Currently, most studies have designed many effective methods based on the proposed DNB theory. For example, Professor Ling Fei’s research group used scRNA-seq data and localized the critical period of colorectal cancer epithelial cell progression as well as regulatory molecules such as FOS/JUN through DNB [9]; the team also detected the pre-aggregation critical time point of α-synuclein in Parkinson’s disease research via DNB and identified MAPKAPK2 as a potential diagnostic biomarker [10]; the teams of Zhou Caicun and Chen Luonan combined the DNB algorithm with multi-omics to identify pre-metastatic biomarkers in early lung cancer and construct a prediction model [11]. However, due to the influence of sample size and data noise, it still remains challenging to identify the critical state of complex diseases.

The classic DNB method requires multiple samples at each time point for verification. However, most complex diseases have a small sample size, which greatly limits the application of the DNB method. Therefore, many researchers have proposed some single-sample methods to address this difficulty, providing a new technical approach for the accurate identification of the critical state of complex diseases. Liu et al. (2022) proposed the Local Network Entropy (LNE) method: by constructing a PPI network to calculate the LNE score of a single sample relative to a healthy reference, they successfully identified the pre-disease states of 10 types of cancers. They also proposed prognostic biomarkers including O-LNE and P-LNE, and explored “dark genes” [12]. Liu and Gao (2018) constructed a pathway network based on differential entropy, analyzed the changes in pathway entropy of diabetic model rats, and the screened differential entropy pathways showed excellent performance in distinguishing diabetic samples from normal samples (with most AUC values > 0.9), which was also verified by an independent dataset [13]. Zhong et al. (2020) proposed the single-sample Kullback-Leibler divergence (sKLD) method: by quantifying the perturbation of a single sample against the background of healthy samples, they successfully located the critical time point of acute lung injury and the critical stages of 5 types of cancers, with significant differences in patient survival before and after the critical state [14]. These methods break through the multi-sample limitation of traditional DNB and provide key tools for the early warning of complex diseases.

More recent studies have further extended single-sample and distributional early-warning frameworks. Liu, Hou, and Feng proposed the Local Network Wasserstein Distance (LNWD) method, which combines local PPI-network structure with Wasserstein distance to quantify sample-specific perturbations during complex disease progression [15]. Zhong et al. developed the sample-perturbed Gaussian graphical model (sPGGM), which embeds Gaussian graphical modeling and PPI prior knowledge into an optimal-transport framework to identify pre-disease stages and signaling molecules at the sample or cell level [16]. These studies show that network-constrained distributional modeling and optimal transport are becoming increasingly relevant for single-sample critical-state detection. In parallel, recent work on multimodal medical reasoning has emphasized that algorithmic outputs should be evaluated for whether they are grounded in the relevant medical evidence rather than only for apparent predictive accuracy [17]. This broader point is consistent with our use of multiple lines of supporting evidence, including stage-wise score patterns, survival-analysis consistency, dynamic molecular-network changes, and biological evidence, when interpreting dCor-LNWD results.

In previous studies, researchers have used the Kullback-Leibler divergence to measure the difference between two distributions, thereby quantifying the statistical perturbation caused by diseased samples. However, the Kullback-Leibler divergence has obvious shortcomings: on the one hand, it cannot normally measure the difference between non-overlapping distributions; on the other hand, it is an asymmetric divergence itself. Although the JS divergence is symmetric (belonging to symmetric divergences), it still requires the premise that there is overlap between distributions. Specifically, for non-overlapping distributions, the calculation result of the KL divergence tends to be positive infinity [18], while the calculation result of the JS divergence tends to a certain fixed constant [19]. In this study, we used the Wasserstein distance to measure differences between local-network probability distributions. The underlying logic of this distance is derived from optimal transport theory, and it provides a natural way to quantify distributional displacement even when the probability mass is redistributed over different parts of the local network. We regard this property as the methodological motivation for using Wasserstein distance in dCor-LNWD, and the empirical comparison with KL-based and entropy-based baselines is reported in the supplementary material. Wasserstein distance has also become an important tool in fields such as machine learning, statistics, and computer vision [20–22].

In addition, most researchers use the Pearson correlation coefficient to measure the correlation between the expression levels of different genes. However, the Pearson correlation coefficient can only characterize the linear correlation between two variables, thus ignoring the potential non-linear correlation between gene expression levels [23]. In this study, we propose to use the distance correlation coefficient to measure the degree of association between the expression levels of two genes. The distance correlation coefficient measures the degree of association by means of the distance matrix of variables. Its core advantage lies in breaking the strong dependence of the traditional Pearson correlation coefficient on linear relationships and normal distribution, and being able to capture associations of any form between variables. At the same time, this coefficient has useful distribution-free properties for evaluating complex dependence patterns in gene-expression data [24–27].

This study proposes a single-sample model-free method for detecting the critical state of complex diseases (see Fig 1(b)), namely the Local Network Wasserstein Distance method based on Distance Correlation (dCor-LNWD). The method evaluates disease-associated perturbations at the level of individual diseased samples by comparing local-network distributions before and after incorporating each diseased sample into the normal reference group. Specifically, the dCor-LNWD method uses normal samples as the reference group. For each diseased sample, a mixed group is generated by adding this sample to the normal group. The screened differential genes are then mapped to the Protein-Protein Interaction (PPI) network, and the local-network probability distributions are constructed using distance correlations between hub genes and neighboring genes. The perturbation introduced by the diseased sample is quantified by calculating the Wasserstein distance between the normal reference distribution and the corresponding mixed-group distribution. This method was applied to four TCGA datasets: Esophageal Carcinoma (ESCA), Kidney Clear Cell Carcinoma (KIRC), Kidney Papillary Cell Carcinoma (KIRP), and Lung Adenocarcinoma (LUAD); as well as a type 2 diabetes dataset in rat adipose tissue (GSE13268) from the GEO database. The detected critical states were further assessed using survival analysis as a clinical consistency check and dynamic molecular-network changes as supporting transcriptomic evidence.

## Materials and methods

### Theoretical background

The dCor-LNWD method is constructed based on the Dynamical Network Biomarker (DNB) theory, which has a rigorous theoretical proof. The DNB theory points out that when a system network approaches a critical state, characteristics such as internal correlation and volatility of the network will undergo significant changes. Among all observed variables, a group of dominant molecules will appear; this group of molecules is called the DNB group. When approaching the critical state, this molecular group must satisfy the following three conditions: first, the volatility of the molecules within the DNB group increases significantly; second, the correlation between the molecules within the group is significantly enhanced; third, the correlation between the molecules within the group and other molecules in the system weakens. More generally, early-warning theory indicates that critical-transition signals reflect loss of stability rather than disease severity itself [28]. In DNB theory, the leading molecular group is expected to show a sharp instability-related signal near the transition point, including increased fluctuation and strengthened within-group association [7,29]. Therefore, dCor-LNWD should be interpreted as an instability-oriented network perturbation index rather than a monotonic measure of late-stage disease burden. After a transition has occurred, the system may settle into another relatively stable disease state, so the strongest warning signal is not necessarily expected to occur at the most advanced or most active disease stage [6,12].

For the calculation of the distance correlation coefficient, let the actually observed sample data be $X=(X_{1},X_{2},\ldots ,X_{n})$ and $Y=(Y_{1},Y_{2},\ldots ,Y_{n})$ respectively. The expression for the sample distance correlation coefficient $dCor_{n}(X,Y)$ is:


$$\begin{array}{c} dCor_{n}(X,Y)=\left\{ \begin{array}{cc} \frac{V_{n}(X,Y)}{S_{n}(X)\cdot S_{n}(Y)} & \text{if }S_{n}(X)>0\text{ and }S_{n}(Y)>0,\\ 0 & \text{otherwise}, \end{array}\right. \end{array}$$ (1)

where the sample distance covariance $V_{n}(X,Y)$ is calculated based on the doubly centered distance matrices $C_{X}$ and $C_{Y}$:


$$\begin{array}{c} V_{n}(X,Y)={\left(\frac{1}{n^{2}}\sum_{i=1}^{n}\sum_{j=1}^{n}C_{X}(i,j)\cdot C_{Y}(i,j)\right)}^{1/2}, \end{array}$$ (2)

in which $C_{X}(i,j)$ and $C_{Y}(i,j)$ are the doubly centered elements in the sample distance matrices $C_{X}$ and $C_{Y}$ respectively. The sample distance standard deviation $S_{n}(X)$ is essentially an autocovariant form of the sample distance covariance, i.e.,:


$$\begin{array}{c} S_{n}(X)=V_{n}(X,X)={\left(\frac{1}{n^{2}}\sum_{i=1}^{n}\sum_{j=1}^{n}C_{X}(i,j{)}^{2}\right)}^{1/2}. \end{array}$$ (3)

The Wasserstein distance is a core metric method for measuring the difference between two distributions, whose core idea is the minimum amount of work required to transform one distribution into another. In this study, the metric constructed by the Wasserstein distance is used to identify the critical state of complex diseases. For two distributions *P* and *Q*, the Wasserstein distance is defined as:


$$\begin{array}{c} W(P,Q)=\underset{\gamma \sim \Pi (P,Q)}{\inf}{𝔼}_{x,y\sim \gamma }\left[\|x-y\|\right], \end{array}$$ (4)

where Π(P,Q) denotes the set of all joint distributions between *P* and *Q*, and *x* and *y* represent sample pairs drawn from a certain joint distribution γ; among the expected values obtained under all joint distributions, the minimum value is the desired Wasserstein distance. In the actual calculation process, we convert it into the solution of the optimal transportation problem [30]. Let $P=\left(p_{1},p_{2},\cdots ,p_{m}\right)$ be the production volume of production sites, and $Q=\left(q_{1},q_{2},\cdots ,q_{n}\right)$ be the demand volume of sales sites, thereby constructing a linear programming problem:


$$\begin{array}{cc} & \min\sum_{i=1}^{m}\sum_{j=1}^{n}c_{ij}f_{ij}\\ & \text{s.t.}\;\left\{ \begin{array}{c} \sum_{j=1}^{n}f_{ij}=p_{i},\\ \sum_{i=1}^{m}f_{ij}=q_{j},\\ f_{ij}\geq 0, \end{array}\right. \end{array}$$ (5)

where $f_{ij}$ represents the shipment volume from production site $p_{i}$ to sales site $q_{j}$, and $c_{ij}$ represents the corresponding transportation cost.

### Method and process

Based on the DNB theory, we take TCGA data as an example to present the specific implementation steps of the dCor-LNWD method:

In the dCor-LNWD framework, normal samples were used to establish the reference local-network distribution. For each individual diseased sample, a mixed group was constructed by adding this sample to the normal reference group. The difference between the normal reference distribution and the corresponding mixed-group distribution was then quantified as the sample-wise dCor-LNWD score.

[Step 1] First, obtain TCGA data and perform gene differential analysis using TCGA counts data. Then, map the screened differential genes to the Protein-Protein Interaction (PPI) network, select interaction edges with a confidence level between genes exceeding 0.800, remove isolated nodes without connections, and finally construct a global network $N^{G}$.
[Step 2] Perform a $\log_{2}$ transformation on the TCGA TPM data, complete data staging according to clinical information, and then map the data processed as above to the generated global network $N^{G}$.
[Step 3] Construction of local network probability distribution. For each gene $g^{k}$, extract the local network $N^{k}\;(k=1,2,\cdots ,L)$ from the global network $N^{G}$, where $\left\{g_{1}^{k},g_{2}^{k},\cdots ,g_{M}^{k}\right\}$ are the first-order neighboring genes of $g^{k}$. For normal group samples, the construction method of the probability distribution corresponding to this local network is:

$$\begin{array}{c} p_{i}^{n}(t)=\frac{\left|{\text{dCor}}^{n}\left(g_{i}^{k}(t),g^{k}(t)\right)\right|}{\sum_{j=1}^{M}\left|{\text{dCor}}^{n}\left(g_{j}^{k}(t),g^{k}(t)\right)\right|}, \end{array}$$ (6)

where the constant *M* represents the number of first-order neighboring genes of gene $g^{k}$, and ${\text{dCor}}^{n}\left(g_{i}^{k}(t),g^{k}(t)\right)$ refers to the distance correlation coefficient between the hub gene $g^{k}$ and its neighboring gene $g_{i}^{k}$ based on *n* normal sample data at time point *t*. Similarly, for the mixed group constructed by adding one individual diseased sample to the normal reference group, the local network probability distribution is:


$$\begin{array}{c} p_{i}^{n+1}(t)=\frac{\left|{\text{dCor}}^{n+1}\left(g_{i}^{k}(t),g^{k}(t)\right)\right|}{\sum_{j=1}^{M}\left|{\text{dCor}}^{n+1}\left(g_{j}^{k}(t),g^{k}(t)\right)\right|}, \end{array}$$ (7)

The distance correlation between the hub gene and each neighboring gene was used as a local dependence weight because distance correlation measures the magnitude of both linear and nonlinear associations. By normalizing these non-negative weights within each local network, we obtained a discrete distribution over the neighboring genes. This distribution represents the relative allocation of the total local association strength around the hub gene, rather than a biochemical reaction probability.

[Step 4] Calculation of local network dCor-LNWD score. Let $P=\left(p_{1}^{n}(t),p_{2}^{n}(t),\cdots ,p_{m}^{n}(t)\right)$ be the probability distribution corresponding to normal group samples, and $Q=\left(p_{1}^{n+1}(t),p_{2}^{n+1}(t),\cdots ,p_{m}^{n+1}(t)\right)$ be the probability distribution corresponding to mixed group samples. The approximate value of the Wasserstein distance is obtained by solving the linear programming problem:

$$\begin{array}{c} W^{k}(P,Q)=\min\sum_{i=1}^{M}\sum_{j=1}^{M}c_{ij}f_{ij} \end{array}$$ (8)

where the cost is approximated by calculating the difference between the expression levels of the two genes:


$$\begin{array}{c} c_{ij}=\left|{\overset{―}{g}}_{i}^{k}(t)-{\overset{―}{g}}_{j}^{k}(t)\right| \end{array}$$ (9)

This cost was used as a baseline expression-space distance between two neighboring genes in the normal reference state. Therefore, redistributing probability mass between genes with similar normal expression levels has a smaller cost, whereas redistributing mass between genes with larger baseline expression differences has a higher cost. Using the normal reference group to define the cost matrix keeps the support geometry fixed across samples, so that the Wasserstein score mainly reflects the redistribution of local association strength induced by the diseased sample. Here, ${\overset{―}{g}}_{i}^{k}(t)$ and ${\overset{―}{g}}_{j}^{k}(t)$ represent the average expression levels of gene $g_{i}^{k}$ and gene $g_{j}^{k}$ based on *n* normal group samples, respectively. $W^{k}(P,Q)$ is used as the dCor-LNWD score of the k-th local network to measure the statistical perturbation introduced by the individual diseased sample relative to the normal reference samples in this local network.

[Step 5] Calculation of global network dCor-LNWD score. According to the above algorithm, first solve the local network dCor-LNWD scores of differential genes at each time point; then sort the local network dCor-LNWD scores of differential genes in each period. To avoid confusion with *n*, which denotes the number of normal reference samples in Step 3, the number of selected top-ranked genes is denoted as *K*. In this study, *K* was fixed at 20 for all datasets. For each stage *t*, the top-*K* gene set $T_{K}(t)$ was obtained from the ranked local-network scores. The shared leading perturbation set $S_{K}$, defined as the intersection of the top-*K* sets across stages, was then used to calculate the global network score so that stage-wise comparisons were based on the same genes:

$$\begin{array}{c} S_{K}=\underset{t}{⋂}T_{K}(t), \end{array}$$ (10)

$$\begin{array}{c} W(t)=\frac{1}{\left|S_{K}\right|}\sum_{g^{k}\in S_{K}}W^{k}(t). \end{array}$$ (11)

Here, $W^{k}(t)$ represents the local dCor-LNWD score of gene $g^{k}$ at stage *t*, and W(t) represents the global network score at that stage. The value *K* = 20 was selec*t*ed as a fixed leading-subset size to focus on the most strongly perturbed local networks while avoiding dataset-specific tuning. To evaluate whether the critical-state calls depended on this parameter, we repeated the calculation using nearby values *K* = 15, 20, and 25. The identified critical stages were unchanged across all five datasets; the sensitivity-analysis results are provided in Supplementary Tables S1 and S2 in S1 File.

We also performed a side-by-side baseline comparison with an sKLD/KL-divergence analysis and an LNE analysis. For the sKLD/KL baseline, the local-network perturbation was quantified using KL-divergence scores calculated from the same stage-specific local-network framework. For the LNE baseline, the entropy change of each local-network probability distribution relative to the normal reference distribution was calculated. To keep the comparison at the same stage-decision level, each method was summarized by the average score of the top 20 genes within each stage, and the predicted critical state was defined as the stage with the largest stage-wise score. For the four TCGA cancer datasets, we further evaluated the survival separation induced by each method-predicted stage boundary by comparing samples before the predicted critical stage with samples at and after that stage. This survival-boundary analysis was treated as a clinical consistency assessment, not as independent validation of a biological tipping point. The detailed comparison is provided in Supplementary Tables S3–S7 in S1 File.

Based on the theoretical background above, a high dCor-LNWD score indicates that the local-network distribution of a disease stage has undergone a strong instability-related perturbation relative to the normal reference state. The stage with the maximum global dCor-LNWD score was therefore used as an operational decision rule for selecting the candidate critical stage, namely the observed stage with the strongest critical-transition signal (see Fig 1(c)). This criterion does not imply that the exact biological tipping point is proven to occur at a single sampled stage, nor that the score must increase monotonically with disease severity. Rather, it identifies the most prominent network-warning signal among the discrete stages available in the dataset; the interpretation was further considered together with the top-*K* sensitivity analysis, survival-analysis consistency, dynamic molecular-network changes, and supporting biological or clinical evidence.

### Data processing

Four clinical tumor datasets were downloaded from the TCGA database (available at: https://portal.gdc.cancer.gov/), namely Esophageal Carcinoma (ESCA), Kidney Clear Cell Carcinoma (KIRC), Kidney Papillary Cell Carcinoma (KIRP), and Lung Adenocarcinoma (LUAD); the dataset GSE13268 was downloaded from the GEO database (available at: https://www.ncbi.nlm.nih.gov/geo/).

The four datasets from TCGA include RNA sequencing data of tumor samples and adjacent non-tumor samples. Tumor samples were staged based on the clinical information provided by TCGA, and samples without staging information were excluded; adjacent non-tumor samples served as reference samples. For the GSE13268 dataset from the GEO database, samples without staging information were also excluded. Meanwhile, according to the gene annotation information provided by the platform, the gene probe names in the expression matrix were converted to gene names. If a gene was mapped to multiple probes, the average value of the expression levels of these probes was taken as the expression level of that gene, while data corresponding to probes without gene annotation information were excluded. Finally, normal group samples and disease group samples from all datasets were obtained.

For the TCGA cancer datasets, all case samples were clinically diagnosed tumor samples grouped by pathological stage; therefore, the state identified by dCor-LNWD was interpreted as a stage-wise critical state during cancer progression.

The normal reference sample sizes were 7 for ESCA, 38 for KIRC, 20 for KIRP, and 29 for LUAD. For the time-series GSE13268 dataset, five control samples were available at each time point. Because the reference groups were relatively small in some datasets, especially ESCA and GSE13268, this potential source of instability was considered when interpreting the results.

In this study, differential analysis of diseased samples at each stage was performed using the R packages edgeR, limma, and DESeq2. Differentially expressed genes (DEGs) were identified using a unified criterion of $|\log_{2}FC|>2$ and an FDR-adjusted *P*-value < 0.05. For each disease stage, genes satisfying this criterion in edgeR, limma, and DESeq2 were intersected, and the union of stage-specific DEGs was used as the final DEG set. After completing the differential analysis of genes, the obtained DEGs were mapped to the Protein-Protein Interaction (PPI) networks of Homo sapiens (humans) and mice (available at: https://cn.string-db.org/). Interaction relationships with a significance level less than 0.800, as well as isolated nodes without interaction relationships, were screened out, and finally the interaction relationships among the DEGs were obtained. The molecular interaction network was visualized using Cytoscape (available at: https://cytoscape.org/).

For the four TCGA cancer datasets, survival analysis was performed as a clinical consistency assessment of the stage boundaries indicated by dCor-LNWD. Overall survival (OS) was used as the endpoint. OS time and event status were obtained from the TCGA survival files and modeled using Surv(OS.time, OS) in the R survival package. Kaplan–Meier curves were fitted with survfit, and differences between stage groups were assessed using the log-rank test as implemented by ggsurvplot in the R survminer package. In the supplementary baseline comparison, unadjusted Cox models were also used to estimate hazard ratios for samples at and after each method-predicted critical stage relative to samples before that stage. These analyses were unadjusted; age and other clinical covariates were not included in a multivariable Cox model because the survival curves were used as an external clinical consistency check rather than as an independent prognostic model or independent proof of a biological tipping point.

## Results

In the research methods section, this paper elaborates on the implementation process of the dCor-LNWD method in detail. To evaluate the applicability of this method, the study applied it to two types of datasets: four cancer datasets from the TCGA database and a type 2 diabetes dataset of rat adipose tissue from the GEO database. The identified critical states were then interpreted together with clinical consistency from survival analysis, dynamic changes in dCor-LNWD scores in the PPI network, and distance-correlation changes between molecular gene-expression levels.

### Identify critical states during cancer progression

The dCor-LNWD method in this study was applied to four cancer datasets from the TCGA database. First, the study staged the cancer data based on clinical information: KIRP, KIRC, and ESCA were divided into four stages (I, II, III, and IV), while LUAD was divided into seven stages (IA, IB, IIA, IIB, IIIA, IIIB, and IV), with each stage corresponding to a group of disease samples. Subsequently, the global dCor-LNWD score of each sample was calculated, and the final score for the corresponding stage was obtained by averaging the scores of all samples. The study plotted a score change curve by stage; when the score in the curve reached the highest point, it indicated that this stage was the stage-wise critical state during cancer progression. To assess clinical consistency with the identified stage boundary, the study plotted survival curves for samples before the identified stage and samples at or after that stage. A higher survival rate before the identified stage was interpreted as consistency with the clinical stage boundary, rather than as independent proof that the stage was a genuine biological tipping point. At the same time, the study also plotted dynamic change diagrams of the molecular network; when significant changes occurred in the network diagram, it indicated that this stage was the critical stage during cancer progression.

**Fig 2:**
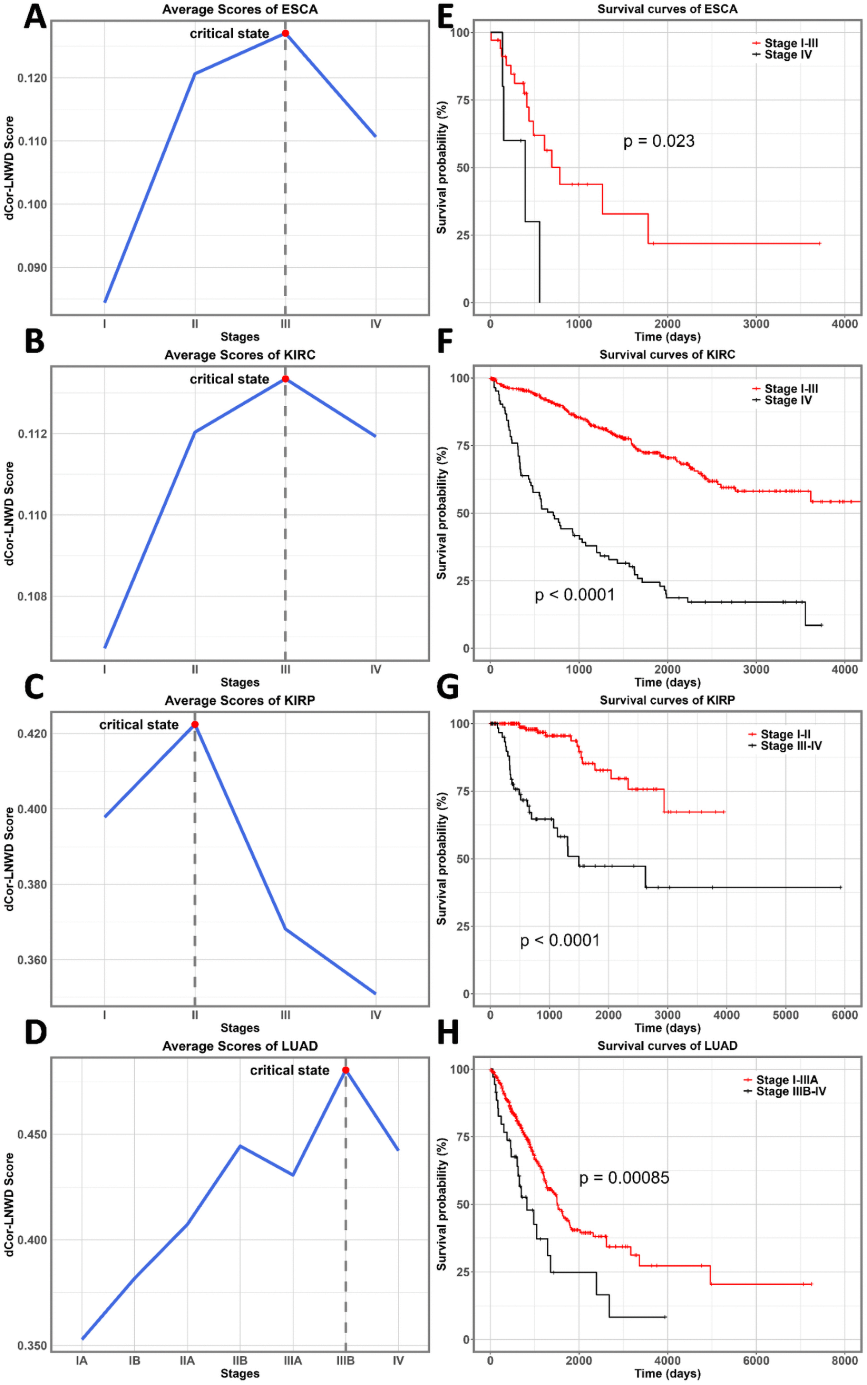
Subfigures A, B, C, and D respectively present the dCor-LNWD score curves of ESCA, KIRC, KIRP, and LUAD; Subfigures E, F, G, and H respectively show the corresponding survival curves for ESCA, KIRC, KIRP, and LUAD, comparing samples before and at/after the identified critical stage. Panels E–H show unadjusted Kaplan–Meier overall-survival curves with log-rank *P*-values.

**Fig 3:**
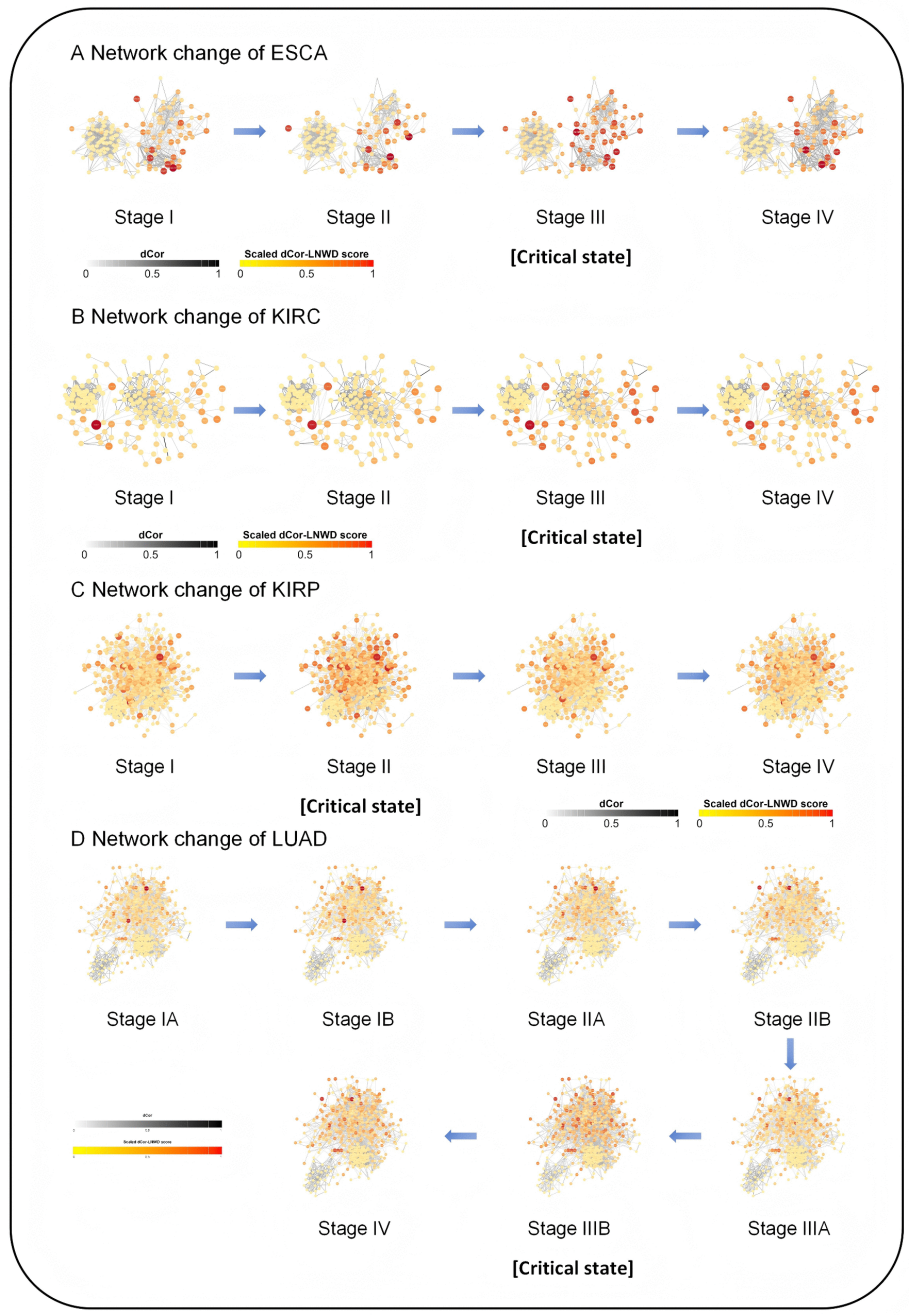
Subfigures A, B, C, and D respectively present the dynamic change diagrams of the molecular networks of ESCA, KIRC, KIRP, and LUAD. Among them, the points represent the dCor-LNWD scores of the corresponding genes in the corresponding period, and the lines between the points represent the distance correlation coefficients between the corresponding gene expression levels.

For esophageal cancer (ESCA), a total of 283 differential genes were screened out through differential analysis. The sample size of the normal group was 7, and the sample sizes of the disease groups corresponding to stage I, II, III, and IV were 15, 75, 56, and 9 respectively. By observing the score curve (see Fig 2A), it can be seen that the dCor-LNWD score changed significantly at stage III, indicating that stage III is the critical stage during ESCA progression. A study by Jeon et al. [31] showed that esophageal cancer exhibits significant changes in treatment response and survival outcomes from stage II to stage III, suggesting that stage III is a clinically relevant critical stage during ESCA progression. More active intervention measures such as perioperative chemotherapy or triple therapy are required to improve the overall survival rate of patients. The survival analysis curve (see Fig 2E) shows that the survival rate before the critical state is significantly higher than that after the critical state, with a significance P-value of 0.023. Meanwhile, by observing the dynamic change diagram of the molecular network (see Fig 3A), it was found that at stage III, both the dCor-LNWD score of the molecular network and the correlation coefficient between molecules changed significantly. Taken together, the stage-wise score pattern, survival consistency, molecular-network changes, and clinical evidence support the interpretation that stage III is the critical stage during ESCA progression.

For clear cell renal cell carcinoma (KIRC), a total of 1,848 differential genes were screened out through differential analysis. The sample size of the normal group was 38, and the sample sizes of the disease groups corresponding to stage I, II, III, and IV were 250, 52, 117, and 73 respectively. By observing the score curve (see Fig 2B), it can be seen that the dCor-LNWD score changed significantly at stage III, indicating that stage III is the critical stage during KIRC progression. A study by DiBianco et al. [32] showed that when stage III renal cell carcinoma invades multiple sites, the cancer-specific mortality rate increases significantly and the prognosis is worse; this clinical evidence is consistent with the critical stage identified in KIRC progression. The survival analysis curve (see Fig 2F) shows that the survival rate before the critical state is significantly higher than that after the critical state, with a significance P-value less than 0.0001. Meanwhile, by observing the dynamic change diagram of the molecular network (see Fig 3B), it was found that at stage III, both the dCor-LNWD score of the molecular network and the correlation coefficient between molecules changed significantly. Taken together, the stage-wise score pattern, survival consistency, molecular-network changes, and clinical evidence support the interpretation that stage III is the critical stage during KIRC progression.

For papillary renal cell carcinoma (KIRP), a total of 1,695 differential genes were screened out through differential analysis. The sample size of the normal group was 20, and the sample sizes of the disease groups corresponding to stage I, II, III, and IV were 162, 20, 46, and 12 respectively. By observing the score curve (see Fig 2C), it can be seen that the dCor-LNWD score changed significantly at stage II, indicating that stage II is the critical stage during KIRP progression. A study by Basile et al. [33] showed that type II papillary renal cell carcinoma differs significantly from type I in pathological features and prognosis; starting from stage II, it exhibits higher invasiveness and worse survival outcomes, suggesting that stage II is a clinically relevant critical stage during KIRP progression. More active clinical management and treatment strategies should be adopted for this stage to improve patients’ prognosis. By observing the survival analysis curve (see Fig 2G), it was found that the survival rate before the critical state was significantly higher than that after the critical state, with a significance P-value less than 0.0001. By observing the dynamic change diagram of the molecular network (see Fig 3C), it was found that at stage II, both the dCor-LNWD score of the molecular network and the correlation coefficient between molecules changed significantly. Taken together, the stage-wise score pattern, survival consistency, molecular-network changes, and clinical evidence support the interpretation that stage II is the critical stage during KIRP progression.

For lung adenocarcinoma (LUAD), a total of 1,853 differential genes were screened out through differential analysis. The sample size of the normal group was 29, and the sample sizes of the disease groups corresponding to stage IA, IB, IIA, IIB, IIIA, IIIB, and IV were 123, 135, 47, 68, 67, 10, and 25 respectively. By observing the score curve (see Fig 2D), it can be seen that the dCor-LNWD score changed significantly at stage IIIB, indicating that stage IIIB is the critical stage during LUAD progression. A study by Jett et al. [34] showed that lung adenocarcinoma exhibits a significant turning point in treatment strategies and survival prognosis at stage IIIB, suggesting that stage IIIB is a clinically relevant critical stage during LUAD progression. Timely intervention through active comprehensive treatment measures such as combined radiotherapy and chemotherapy is required to improve patients’ survival outcomes. By observing the survival analysis curve (see Fig 2H), it was found that the survival rate before the critical state was significantly higher than that after the critical state, with a significance P-value of 0.00085. By observing the dynamic change diagram of the molecular network (see Fig 3D), it was found that at stage IIIB, both the dCor-LNWD score of the molecular network and the correlation coefficient between molecules changed significantly. Taken together, the stage-wise score pattern, survival consistency, molecular-network changes, and clinical evidence support the interpretation that stage IIIB is the critical stage during LUAD progression.

### Identify the critical state of type 2 diabetes in rats

The dCor-LNWD method was applied to the microarray dataset (GSE13268) of type 2 diabetes in rat adipose tissue. At each time point, five control samples and five experimental samples were collected, so each time point contained 10 adipose-tissue samples. The control and experimental groups were Goto-Kakizaki (GK) rats fed with a normal diet and a high-fat diet, respectively. A total of 50 rats were sacrificed at 4, 8, 12, 16, and 20 weeks, yielding 50 adipose-tissue samples. After differential analysis of the microarray data, a total of 283 differential genes were obtained.

**Fig 4:**
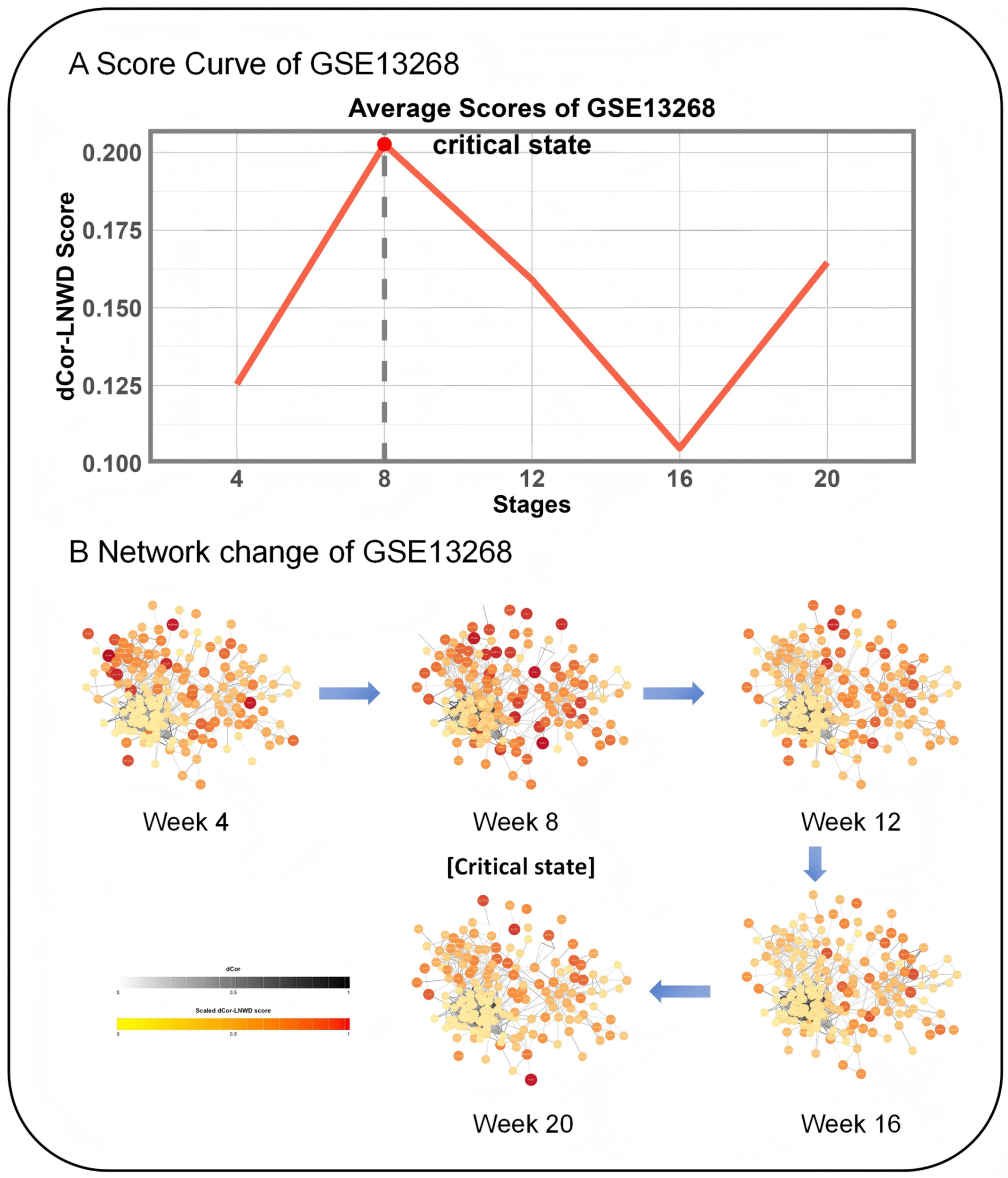
Subgraph A shows the dCor-LNWD score curve of the GSE13268 dataset, and Subgraph B shows the dynamic change graph of the distance correlation coefficient between the dCor-LNWD score of genes and gene expression levels in the PPI network.

By observing the dCor-LNWD score curve (see Fig 4A), it was found that the score reached the highest point at the 8th week, indicating that this period was the critical state of type 2 diabetes in rats. A study by Almon et al. [35] showed that the insulin level of GK rats gradually decreased after the 8th week; by the 20th week, the insulin level of GK rats was lower than that of the control group, which indicated that the condition of diabetes in GK rats began to worsen after the 8th week, consistent with our detection results. By observing the dynamic change diagram of the molecular network (Fig 4B), it was found that the molecular network changed significantly at the 8th week, providing additional transcriptomic support for the identified critical state.

We further performed a sensitivity analysis for the top-*K* gene parameter used in the global score calculation. When *K* was varied around the fixed value used in the main analysis (*K* = 15, 20, and 25), the identified critical stages remained unchanged for all five datasets: ESCA stage III, KIRC stage III, KIRP stage II, LUAD stage IIIB, and GSE13268 week 8. These results indicate that the reported critical-state calls were not driven by a single arbitrary choice of the top-*K* parameter within this range (Supplementary Tables S1 and S2 in S1 File).

In addition, we compared dCor-LNWD with sKLD/KL-divergence and LNE baseline analyses on the same five datasets. Using the maximum stage-wise top-20 average score as the decision rule, dCor-LNWD identified the same critical states supported by the main analysis in all five datasets. The sKLD/KL baseline agreed for LUAD and GSE13268, whereas the LNE baseline agreed for KIRP, LUAD, and GSE13268. For the four TCGA cancer datasets, the dCor-LNWD-predicted stage boundaries produced significant overall-survival separation in all four cancers, with log-rank P≤0.0009 and hazard ratios greater than 2 for the at/after-critical groups. Some baseline-predicted boundaries also produced significant survival separation, especially when the boundary involved late stages; however, the sKLD/KL peak for KIRP was not estimable as a before-versus-after survival split because it occurred at stage I, and some baseline peaks corresponded to terminal stage-IV boundaries. The complete stage-wise scores, peak-stage comparison, and survival-boundary clinical-consistency assessment are reported in Supplementary Tables S3–S7 in S1 File. Because independent binary labels for critical and non-critical stages were not available, these results were interpreted as side-by-side stage-score and clinical-consistency comparisons rather than as a comprehensive benchmark based on classification metrics such as AUC, sensitivity, or specificity.

## Discussion

Identifying early warning signals before disease progression and thereby implementing timely interventions is of great significance for preventing or delaying disease advancement. However, due to problems such as insufficient sample size in most complex diseases, it is quite challenging to accurately identify the pre-disease or critical transition state under the condition of a small number of samples. This study proposes a single-sample model-free method (dCor-LNWD method), which incorporates a single diseased sample into the normal group to form a mixed group. It constructs the probability distribution of the network by calculating the distance correlation coefficient between local network hub genes and neighboring genes, then uses the Wasserstein distance to measure the distribution difference between the normal group and the diseased sample, and finally evaluates the statistical perturbation caused by the diseased sample.

One limitation of this study is that the number of normal reference samples was relatively small in some datasets. This issue is most evident for ESCA, which contained seven adjacent non-tumor samples, and for the GSE13268 time-series data, which contained five control samples at each time point. Although dCor-LNWD is designed for sample-wise analysis under small-sample conditions, the normal reference local-network distribution is still estimated from the available normal samples. A smaller reference group may increase the variability of the estimated distance correlations, the cost matrix, and the resulting Wasserstein-based perturbation scores, which could affect the stability of the stage-wise score curve. Therefore, the critical-state calls were interpreted together with several supporting observations, including stage-wise score peaks, survival differences in the TCGA cohorts, dynamic molecular-network changes, and consistency with previous clinical or biological evidence. Future studies using larger normal reference cohorts, independent external datasets, or resampling-based stability assessment will help further evaluate the robustness of dCor-LNWD.

The dCor-LNWD method constructed in this study was applied to four cancer datasets from the TCGA database and one rat type 2 diabetes dataset from the GEO database, and the corresponding critical states were successfully identified. For example, stage III was identified as the critical stage during ESCA progression, which requires timely interventions such as surgery to prevent further disease progression; stage III was identified as the critical stage during KIRC progression, after which renal cell carcinoma begins to invade multiple sites and the cancer mortality rate increases significantly; stage II was identified as the critical stage during KIRP progression, after which the cell carcinoma shows higher invasiveness and worse survival outcomes; week 8 was identified as the critical time point in the progression of type 2 diabetes in rats, at which time the insulin level of rats begins to decrease and the condition of diabetes begins to worsen.

For the TCGA cancer cohorts, these identified stages should be interpreted as critical stages within the progression of already diagnosed cancers, rather than premalignant or cancer-free pre-onset states. Validation of true pre-cancer states would require premalignant, longitudinal, or early-screening cohorts.

The critical-state interpretation in this study was supported by previous clinical or biological evidence, stage-wise score patterns, molecular-network changes, and survival-analysis consistency. For the cancer datasets in TCGA, survival curves before and at/after the identified stage showed the expected prognosis separation, but this was interpreted only as a clinical consistency assessment because later cancer stages are generally associated with poorer survival. In addition, the study plotted dynamic molecular-network diagrams for the four cancer datasets and one rat type 2 diabetes dataset. In the molecular networks corresponding to the identified critical states, both the dCor-LNWD scores and the distance correlation coefficients between the expression levels of corresponding genes changed significantly, providing supporting transcriptomic evidence for the identified stages.

The method proposed in this study is designed to support sample-wise critical-state assessment under limited-sample conditions. The use of distance correlation allows dCor-LNWD to evaluate both linear and nonlinear dependence patterns between gene-expression profiles, while the Wasserstein distance provides a way to measure redistribution of local-network probability mass relative to the normal reference state. In the additional side-by-side analysis, dCor-LNWD showed the highest agreement with the critical stages supported by the integrated supporting evidence among the compared methods on these five datasets, and its predicted cancer-stage boundaries were consistently associated with significant overall-survival separation. Nevertheless, survival separation across cancer stages should not be interpreted as independent validation of a biological tipping point, because later pathological stages are generally associated with poorer prognosis. Independent validation using external cohorts, longitudinal samples, orthogonal biomarker measurements, or transcriptomic trajectory data will be needed to further confirm the biological tipping-point interpretation.

## Conclusion

This study proposes a single-sample critical state identification method for complex diseases based on the distance correlation coefficient. This method can measure both linear and nonlinear associations between gene-expression profiles and quantify local-network perturbations at the individual-sample level, thereby supporting critical-state detection in complex disease datasets. Therefore, it has important application potential in the fields of personalized disease diagnosis and prevention.

## Supporting information

S1 File Supplementary material. Top-*K* sensitivity and baseline comparison analyses.
(PDF)
