# Supplementary material for: Detection of critical transition states in complex diseases based on distance correlation coefficient
Source: PLoS One. 2026 Jul 16;21(7):e0341473. doi: 10.1371/journal.pone.0341473 (PMC13375029; doi:10.1371/journal.pone.0341473)
Supplement: S1 File — Top-K sensitivity and baseline comparison analyses. (PDF) [file pone.0341473.s001.pdf]

Supplementary Material

Top-K Sensitivity and Baseline Comparison Analyses

Supplementary Table S1. Summary of the top-K sensitivity analysis

The dCor-LNWD workflow ranks local networks by their stage-specific local scores. For each value of K, the top-K genes were selected at each stage, and the shared leading set across stages was used to calculate the stage-wise global score. The reported critical stage corresponds to the stage with the largest global score. The results show that the identified critical stages were unchanged when K was varied around the value used in the main analysis ($K = 20$).

Sensitivity analysis for $K = 15$, 20, and 25. Values in parentheses indicate the number of genes in the shared top-K set used for the stage-wise score calculation.

Dataset	$K = 15$	$K = 20$	$K = 25$	Reported critical stage
ESCA	III (10)	III (13)	III (15)	III
KIRC	III (14)	III (17)	III (20)	III
KIRP	II (7)	II (9)	II (12)	II
LUAD	IIIB (7)	IIIB (12)	IIIB (15)	IIIB
GSE13268	8 weeks (2)	8 weeks (3)	8 weeks (3)	8 weeks

Supplementary Table S2. Detailed stage-wise global scores

To further show how the critical stages were obtained, the detailed stage-wise global scores from the sensitivity analysis are listed below. The largest score within each row identifies the predicted critical stage. For readability, scores are rounded to three decimal places.

Detailed sensitivity results for ESCA, KIRC, and KIRP.

Dataset	K	Candidates	Shared	I	II	III	IV
ESCA	15	148	10	0.093	0.132	**0.145**	0.123
ESCA	20	148	13	0.084	0.121	**0.127**	0.111
ESCA	25	148	15	0.079	0.115	**0.119**	0.105
KIRC	15	191	14	0.115	0.122	**0.124**	0.121
KIRC	20	191	17	0.106	0.113	**0.114**	0.112
KIRC	25	191	20	0.099	0.105	**0.106**	0.105
KIRP	15	653	7	0.409	**0.452**	0.391	0.374
KIRP	20	653	9	0.398	**0.422**	0.368	0.351
KIRP	25	653	12	0.368	**0.393**	0.345	0.335

Detailed sensitivity results for LUAD.

Dataset	K	Candidates	Shared	IA	IB	IIA	IIB	IIIA	IIIB	IV
LUAD	15	630	7	0.406	0.425	0.460	0.518	0.504	**0.570**	0.525
LUAD	20	630	12	0.353	0.382	0.407	0.444	0.431	**0.481**	0.442
LUAD	25	630	15	0.335	0.363	0.385	0.416	0.414	**0.451**	0.414

Detailed sensitivity results for GSE13268.

Dataset	K	Candidates	Shared	Week 4	Week 8	Week 12	Week 16	Week 20
GSE13268	15	187	2	0.133	**0.207**	0.130	0.113	0.150
GSE13268	20	187	3	0.125	**0.203**	0.159	0.105	0.165
GSE13268	25	187	3	0.125	**0.203**	0.159	0.105	0.165

Analysis of the top-K sensitivity results

Across all datasets, varying K from 15 to 25 changed the number of genes retained in the shared top-K set, but did not change the stage with the maximum global score. ESCA and KIRC consistently reached their highest scores at stage III, KIRP at stage II, LUAD at stage IIIB, and GSE13268 at week 8. These results indicate that the critical-state identification was robust to nearby choices of the top-K parameter rather than being determined by a single arbitrary value.

Supplementary Table S3. Summary of the baseline comparison

To address whether the dCor-LNWD results were consistent with existing single-sample strategies, we compared the dCor-LNWD stage-wise score profiles with two baseline analyses: an sKLD/KL-divergence analysis and a local network entropy (LNE) analysis. Because strict shared top-20 intersections were not available for every baseline in every dataset, the side-by-side comparison used the average score of the top 20 genes within each stage for each method. The predicted critical stage was defined as the stage with the largest stage-wise score. The dCor-LNWD method recovered the critical state supported in the main analysis for all five datasets, whereas the sKLD/KL and LNE baselines showed partial agreement.

Peak-stage comparison across methods. "Agreement" indicates whether the method identified the same critical stage as the stage supported by the main dCor-LNWD analysis and integrated supporting evidence.

Dataset	Supported critical state	dCor-LNWD	sKLD/KL	LNE
ESCA	III	**III**	IV	II
KIRC	III	**III**	II	IV
KIRP	II	**II**	I	**II**
LUAD	IIIB	**IIIB**	**IIIB**	**IIIB**
GSE13268	8 weeks	**8 weeks**	**8 weeks**	**8 weeks**
Agreement	–	5/5	2/5	3/5

Supplementary Table S4. Detailed baseline scores for four-stage cancer datasets

Stage-wise scores were calculated as the mean of the top 20 local-network scores within each stage for each method. The largest value in each row is shown in bold.

Stage-wise top-20 average scores for ESCA, KIRC, and KIRP.

Dataset	Method	I	II	III	IV	Peak
ESCA	dCor-LNWD	0.0794	0.1063	**0.1112**	0.1005	III
ESCA	sKLD/KL	0.0529	0.0730	0.0699	**1.9972**	IV
ESCA	LNE	0.0142	**0.0184**	0.0183	0.0183	II
KIRC	dCor-LNWD	0.0992	0.1048	**0.1065**	0.1058	III
KIRC	sKLD/KL	0.0352	**0.0382**	0.0380	0.0372	II
KIRC	LNE	0.0189	0.0205	0.0212	**0.0223**	IV
KIRP	dCor-LNWD	0.3445	**0.3617**	0.3334	0.3217	II
KIRP	sKLD/KL	**0.2018**	0.2009	0.1884	0.1856	I
KIRP	LNE	0.0401	**0.0415**	0.0389	0.0388	II

Supplementary Table S5. Detailed baseline scores for LUAD

Stage-wise top-20 average scores for LUAD.

Method	IA	IB	IIA	IIB	IIIA	IIIB	IV	Peak
dCor-LNWD	0.3170	0.3449	0.3656	0.3818	0.3813	**0.4125**	0.3832	IIIB
sKLD/KL	0.1670	0.1789	0.1816	0.1841	0.1883	**0.2033**	0.1838	IIIB
LNE	0.0424	0.0475	0.0485	0.0489	0.0512	**0.0526**	0.0460	IIIB

Supplementary Table S6. Detailed baseline scores for GSE13268

Stage-wise top-20 average scores for GSE13268.

Method	Week 4	Week 8	Week 12	Week 16	Week 20	Peak
dCor-LNWD	0.1286	**0.2129**	0.1217	0.0973	0.1405	8 weeks
sKLD/KL	0.2917	**0.5076**	0.3303	0.3372	0.3982	8 weeks
LNE	0.0122	**0.0217**	0.0129	0.0108	0.0149	8 weeks

Supplementary Table S7. Survival clinical-consistency assessment of method-predicted stage boundaries

For the four TCGA cancer datasets, we further evaluated whether the stage boundary implied by each method produced clinically meaningful overall-survival separation. For each method, sam-

ples before the predicted critical stage were compared with samples at and after the predicted critical stage. Sub-stages in ESCA, KIRC, and KIRP were mapped to the corresponding main pathological stage, whereas LUAD sub-stages were retained. Hazard ratios (HRs) were estimated for the at/after-critical group relative to the before-critical group using an unadjusted Cox model. A boundary predicted at the first stage cannot define a before-critical group and was therefore considered not estimable.

Overall-survival clinical-consistency assessment based on method-predicted critical stages in TCGA cancer datasets.

Dataset	Method	Peak	Grouping	N	Events	Log-rank P	HR (95% CI)
ESCA	dCor-LNWD	III	I–II vs III–IV	95/65	28/35	< 0.0001	3.24 (1.91–5.50)
ESCA	sKLD/KL	IV	I–III vs IV	151/9	55/8	0.0002	3.85 (1.81–8.21)
ESCA	LNE	II	I vs II–IV	18/142	4/59	0.0203	3.22 (1.14–9.07)
KIRC	dCor-LNWD	III	I–II vs III–IV	320/206	56/116	< 0.0001	3.93 (2.85–5.40)
KIRC	sKLD/KL	II	I vs II–IV	263/263	43/129	< 0.0001	3.35 (2.37–4.73)
KIRC	LNE	IV	I–III vs IV	443/83	105/67	< 0.0001	4.58 (3.37–6.24)
KIRP	dCor-LNWD	II	I vs II–IV	170/87	12/27	< 0.0001	4.36 (2.20–8.64)
KIRP	sKLD/KL	I	Not estimable	0/257	0/39	NA	NA
KIRP	LNE	II	I vs II–IV	170/87	12/27	< 0.0001	4.36 (2.20–8.64)
LUAD	dCor-LNWD	IIIB	IA–IIIA vs IIIB–IV	455/35	156/22	0.0009	2.10 (1.34–3.29)
LUAD	sKLD/KL	IIIB	IA–IIIA vs IIIB–IV	455/35	156/22	0.0009	2.10 (1.34–3.29)
LUAD	LNE	IIIB	IA–IIIA vs IIIB–IV	455/35	156/22	0.0009	2.10 (1.34–3.29)

Analysis of the baseline comparison

The baseline comparison showed that the dCor-LNWD score profile identified the supported critical state in all five datasets. The sKLD/KL baseline identified the same peak stage for LUAD and GSE13268, but shifted the peak to stage IV for ESCA, stage II for KIRC, and stage I for KIRP. The LNE baseline agreed with dCor-LNWD for KIRP, LUAD, and GSE13268, but shifted the peak to stage II for ESCA and stage IV for KIRC.

The additional survival-boundary analysis showed that the dCor-LNWD-predicted boundaries produced significant overall-survival separation in all four TCGA cancer datasets, with HRs greater than 2 for the at/after-critical groups. The sKLD/KL and LNE baselines also produced significant survival separation for several datasets, especially when the predicted boundary involved a late stage. However, the sKLD/KL result for KIRP was not estimable because the predicted peak was stage I, leaving no before-critical group, and the sKLD/KL result for ESCA and the LNE result for KIRC corresponded to terminal stage-IV boundaries. These survival results therefore support the clinical consistency assessment while also showing why survival separation alone should not be treated as the sole benchmark for critical-state detection. Overall, the evidence supports dCor-LNWD as the method with the highest concordance with the integrated supporting evidence in these datasets, rather than establishing a universal superiority claim over all existing single-sample methods.
